# Medical students’ resilience: a protective role on stress and quality of life in clerkship

**DOI:** 10.1186/s12909-019-1912-4

**Published:** 2019-12-27

**Authors:** Yung Kai Lin, Chia-Der Lin, Blossom Yen-Ju Lin, Der-Yuan Chen

**Affiliations:** 1 Surgery Department, Chiayi Branch, Taichung Veterans General Hospital, Chiayi, Taiwan, Republic of China; 2 Division of Cardiovascular Surgery, Taichung Veterans General Hospital, Taichung, Taiwan, Republic of China; 3 School of Medicine, China Medical University, Taichung, Taiwan, Republic of China; 4 Department of Otolaryngology-Head & Neck Surgery, China Medical University Hospital, Taichung, Taiwan, Republic of China; 5 Department of Medical Humanities and Social Sciences, School of Medicine, College of Medicine, Chang Gung University, No.259, Wenhua 1st Rd., Guishan Dist, Taoyuan City, 33302 Taiwan, Republic of China; 6 Center of Rheumatology and Immunology, China Medical University Hospital, Taichung, Taiwan, Republic of China; 7 College of Medicine, China Medical University, Taichung, Taiwan, Republic of China

**Keywords:** Workplace stress, Physical demands, Psychological demands, Resilience, Burnout, Compassion satisfaction, Medical students, Clerkship

## Abstract

**Background:**

Resilience refers to the ability to be flexible and adaptive in response to challenges. Medical students in clerkship who are transitioning from medical studies to clinical practice face a variety of workplace demands that can lead to negative learning experiences and poor quality of life. This study explored whether medical students’ resilience plays a protective role against the stresses incurred during workplace training and on their professional quality of life during clerkships.

**Methods:**

This was a 1-year prospective web-based questionnaire study comprising one cohort of medical students in their fifth year who were working as clerks as part of their 6-year medical education programme at one medical school in Taiwan between September 2017 and July 2018. Web-based, validated, structured, self-administered questionnaires were used to measure the students’ resilience at the beginning of the clerkship and their perceived training stress (i.e. physical and psychological demands) and professional quality of life (i.e. burnout and compassion satisfaction) at each specialty rotation. Ninety-three medical students who responded to our specialty rotation surveys at least three times in the clerkship were included and hierarchical regressions were performed.

**Results:**

This study verified the negative effects of medical students’ perceived training stress on burnout and compassion satisfaction. However, although the buffering (protective) effects of resilience were observed for physical demands (one key risk factor related to medical students’ professional quality of life), this was not the case for psychological demands (another key risk factor). In addition, through the changes in R square (∆R^2^) values of the hierarchical regression building, our study found that medical students’ perceived training stresses played a critical role on explaining their burnout but their resilience on their compassion satisfaction.

**Conclusions:**

Medical students’ resilience demonstrated a buffering effect on the negative relationship between physical demands and professional quality of life during clerkships. Moreover, different mechanisms (predictive paths) leading to medical students’ professional quality of life such as burnout and compassion satisfaction warrant additional studies.

## Background

Resilience refers to the ability of people to ‘bounce back’ when they encounter difficulties [1]. These difficulties can range from adversities to traumas, tragedies, and threats; these can even be significant sources of stress, such as family and relationship problems, serious health problems, or workplace and financial stresses [2]. Resilience is viewed as a flexible and adaptive act performed in response to challenges or an aggregate concept of multiple qualities, both of which indicate an ability to survive and address the likely consequences of handling pressure or adversity [3]. Although medical students’ resilience has not been found to be related to their academic course performance [4], the role of resilience has been shown to be positively associated with the well-being of medical students. For instance, previous cross-sectional questionnaire studies have revealed associations between medical students’ resilience and their lower levels of psychological distress [4], better life satisfaction [5, 6], happiness [5], higher quality of life [7], fewer anxiety symptoms [8], and increased subjective well-being [9, 10]. However, one study in Canada revealed that medical students had higher perceived stress, poorer coping skills, and lower resilience than age- and gender-matched peers in the general population [11].

One study argued that medical students face numerous stressors during their study curricula, which require adequate resilience to ensure healthy adaptation [12]. In particular, medical students experience considerable challenges in their learning experiences and career development when they transit from the classroom to clinical environments or progress from the position of junior resident to attending physician. Whether before or after graduation, undergoing clinical training is more challenging than studying at school and can trigger many physical and mental health conditions (particularly mental disorders and emotional exhaustion) in medical students [13]. Studies have also revealed that medical students can encounter stressful clinical events [14] and psychological and physical demands [15, 16] during their clinical training years. A review study covering the peer-reviewed, English-language articles in MEDLINE between 1990 and 2015 revealed a prevalence of burnout among trainees, including medical students, residents, and interns, to a much higher level than found among the general population [17]. Another study indicated that medical students should be equipped with not only medical knowledge but also an awareness of how to properly care for themselves [13].

Resilience is viewed as a way for individuals to manage stress, survive competently, and engage in learning despite mental pressures [18], therefore, our study explored whether medical students’ resilience could play a protective role in workplace training stress and professional quality of life during clerkships. Our specific hypotheses are as follows:

1. Medical students’ workplace training stress is negatively related to their professional quality of life during clerkships.
2. If Hypothesis 1 is true, medical students’ resilience is a protective factor that buffers the negative relationship between their workplace training stress and professional quality of life.

## Methods

This was a 1-year non-experimental, prospective web-based questionnaire study.

### Participants

Our study comprised one cohort of medical students undergoing their clerkships during their fifth year of a 6-year medical education programme at one medical school in Taiwan between September 2017 and July 2018. In Taiwan, medical students undergoing clerkships are equivalent to third- or fourth-year students undergoing clinical training in 4-year graduate medical programmes in western countries. One recruitment meeting of approximately 20 min was held during the students’ pre-clerkship courses, 1 week before the start of their clerkships. Of 199 medical students, 151 (75.9%) agreed to participate in the study and provided written informed consent.

### Instrument development and administration

Structured and self-administered questionnaires were used in this study, consisting of the following measures:

#### Resilience of medical students

Resilience was measured using the 14-item Resilience Scale, with each item evaluated on a 7-point Likert-type scale from 1 (strongly disagree) to 7 (strongly agree) [19]. Principal components factor analysis was performed, and one common factor was constrained. One item (‘self-discipline is important’) with a factor loading of < 0.4 was deleted [20]. A confirmatory factor analysis was then used to verify the items (*p* < .05) with an asymptotically distribution-free bootstrap model [21, 22] for construct validity testing and yielded moderate model fit. The Cronbach’s alpha was 0.874. Finally, the resilience score of 13 items was calculated by the mean for further analysis. Detailed information is listed in Table 1.

**Table 1:** Background information of the medical students (*n* = 93)

Variable	Mean (Freq.)	SD (%)	Confirmatory factor analyses^b^		
			Regression estimation lambda	Critical ratio (CR)	Squared multiple correlation
Demographics					
Sex					
Male	(43)	(46)			
Female	(50)	(54)			
Age (Year)	22.903	0.933			
Resilience^a^ (scale 1~7)	5.494	0.735			
When I am in a difficult situation, I can usually find my way out of it.	5.645	1.007	0.949	8.604	0.688
My belief in myself gets me through hard times.	5.538	1.119	1.000	(Constrained)	0.619
I usually manage one way or another.	6.118	0.640	0.545	7.597	0.561
In an emergency, I am someone people can generally rely on.	5.538	1.175	0.986	7.456	0.544
I am determined.	5.333	1.192	0.853	6.185	0.396
I can get through difficult times because I have experienced difficulty before.	5.398	1.217	0.829	5.846	0.359
My life has meaning.	5.688	1.113	0.754	5.811	0.355
I can usually find something to laugh about.	5.903	1.124	0.693	5.230	0.295
I keep interested in things.	5.215	1.374	0.834	5.138	0.285
I feel that I can handle many things at a time.	4.806	1.505	0.878	4.919	0.264
I usually take things in stride.	4.000	1.460	0.899	5.223	0.294
I fell proud that I have accomplished things in life.	6.151	0.955	0.464	4.032	0.182
I am friends with myself.	6.086	1.049	0.395	3.087	0.110

^a^ One item from the original Resilience Scale (RS-14) by Wagnild [19] was deleted due to having a factor loading of < 0.4

^b^Overall model fit of resilience according to Bollen-Stine χ^2^ = 89.766, df = 65, Normed Chi-sqr (χ^2^/df) = 1.381, GFI = 0.830, AGFI = 0.763, CFI = 0.945, IFI = 0.947, TLI = 0.934, RMSEA = 0.064, and Hoelter’s *N* = 67.766

#### Training stress of medical students in individual specialty rotations

By using Karasek’s Job Demands–Control model of job strain, stress during clinical training was measured through 10 items adapted from the Job Content Questionnaire, with the responses evaluated on a 5-point scale from 1 (strongly disagree) to 5 (strongly agree) [23]. Two stress constructs, namely psychological and physical demands, were extracted [15]. Psychological demands referred to mental workloads such as hard work, fast-paced work environment, busy at work, not enough sleeping time, work fatigue and intense work concentration, whereas physical demands referred to physical loads including physical positions, activities and loads [24]. Factor analysis revealed that the psychological (six items) and physical (four items) demands had factor loadings of 0.74–0.84 and 0.87–0.93, respectively. Confirmatory factor analyses was then used to verify the items (*p* < .05) with an asymptotically distribution-free bootstrap model [21, 22] for construct validity testing and yielded good model fit. The Cronbach’s alpha for psychological and physical demands was 0.903 and 0.942, respectively (Table 2). Detailed information is listed in Table 2. Factor scores for both psychological and physical demands were calculated using a regression model for further analysis [15].

**Table 2:** Medical students’ perceived training stress and professional quality of life during their individual specialty rotations (*n* = 1073)

Variable	Mean (Freq.)	SD (%)	Confirmatory factor analyses^b^		
			Regression estimation lambda	Critical ratio (CR)	Squared multiple correlation
Workplace training stress					
*Psychological demands (scale 1–5)*	2.696	0.883			
Hard work	2.546	1.016	0928	27.737	0.696
Fast-paced work environment	2.754	1.089	0.968	26.730	0.660
Busy at work	2.556	1.045	0931	29.041	0.664
Not enough sleeping time	2.584	1.122	0972	29.394	0.626
Work fatigue	2.589	1.145	1.000	(Constrained)	0.637
Intense work concentration	3.145	1.031	0.718	21.227	0.405
*Physical demands (scale 1–5)*	***1.810***	***0.819***			
Awkward arm position	1.802	0.867	0.965	65.112	0.929
Awkward body position	1.843	0.921	1.000	(Constrained)	0.883
Rapid physical activity	1.870	0.913	0.905	45.307	0.736
Lifting a heavy load	1.725	0.846	0.796	39.772	0.663
Professional quality of life in the rotated specialties^a^					
*Burnout (scale 1–5)*	2.346	0.613			
I have beliefs that sustain me. (R)	2.331	0.967	0.989	37.822	0.758
I am the person I always wanted to be. (R)	2.188	0.980	1	(Constrained)	0.754
I feel trapped by my job as a medical doctor.	2.312	0.925	0.635	19.411	0.342
I am happy. (R)	2.321	0.759	0.587	22.973	0.433
I feel overwhelmed because my work load seems endless.	2.261	0.927	0.531	15.794	0.238
I am a very caring person. (R)	2.609	0.990	0.835	27.575	0.515
I feel connected to others. (R)	2.172	0.844	0.625	22.388	0.398
I feel worn out because of my work as a medical doctor.	2.675	1.029	0.424	10.916	0.123
I feel “bogged down” by the system.	2.670	1.026	0.380	9.803	0.099
I am not as productive at work because I am losing sleep over traumatic experiences of a person I help.	1.915	0.829	0.297	9.552	0.093
*Compassion satisfaction (scale 1–5)*	3.505	0.815			
I am proud of what I can do to help.	3.456	0.985	1	(Constrained)	0.791
My work makes me feel satisfied.	3.598	0.948	0.949	42.249	0.769
I am happy that I chose to do this work.	3.579	0.958	0.952	41.564	0.758
I like my work as a medical doctor.	3.637	0.945	0.938	41.370	0.756
I have happy thoughts and feelings about those I help and how I could help them.	3.751	0.925	0.918	41.668	0.756
I believe I can make a difference through my work.	3.518	0.971	0.958	41.500	0.747
I am pleased with how I am able to keep up with helping techniques and protocols.	3.546	0.991	0.953	39.062	0.711
I get satisfaction from being able to help people.	3.561	0.877	0.838	38.309	0.700
I feel invigorated after working with those I help.	3.663	0.949	0.907	38.534	0.702
I have thoughts that I am a “success” as a medical doctor.	2.744	0.961	0.699	24.502	0.407

^a^The word ‘medical doctor’ replaces the original ‘helper’ in the Chinese-language ProQOL, Version V [25], to better match the medical students’ scenarios. (R) refers to reversing the original score from 1 to 5, 2 to 4, 4 to 2, and 5 to 1

^b^Overall model fit: (1) psychological demands according to Bollen-Stine χ^2^ = 12.137, df = 9, Normed Chi-sqr (χ^2^/df) = 1.349, GFI = 0.997, AGFI = 0.993, CFI = 0.999, IFI = 0.999, TLI = 0.999, RMSEA = 0.018, and Hoelter’s *N* = 808.732; (2) physical demands according to Bollen-Stine χ^2^ = 5.504, df = 2, Normed Chi-sqr (χ^2^/df) = 2.752, GFI = 0.999, AGFI = 0.994, CFI = 0.999, IFI = 0.999, TLI = 0.998, RMSEA = 0.040, and Hoelter’s *N* = 418.776; (3) burnout according to Bollen-Stine χ^2^ = 1518.578, df = 35, Normed Chi-sqr (χ^2^/df) = 43.388, GFI = 0.694, AGFI = 0.519, CFI = 0.698, IFI = 0.699, TLI = 0.612, RMSEA = 0.199, and Hoelter’s *N* = 25.810; and (4) compassion satisfaction according to Bollen-Stine χ^2^ = 50.530, df = 35, Normed Chi-sqr (χ^2^/df) = 1.444, GFI = 0.995, AGFI = 0.993, CFI = 0.999, IFI = 0.999, TLI = 0.998, RMSEA = 0.020, and Hoelter’s *N* = 746.605

#### Medical students’ professional quality of life in individual specialty rotations

The medical students’ professional quality of life during their clerkships was measured through burnout and compassion satisfaction items using the Professional Quality of Life Scale (ProQOL), Version V [25]. The ProQOL contains questions on the positive and negative effects of working with individuals who have experienced stress [26]. The ProQOL is offered in several languages, and the Chinese version was used in this study with one minor modification: the original word ‘helper’ was replaced with ‘medical doctor’ to better match the medical students’ scenarios.

Burnout is a negative emotion associated with feelings of difficulty and hopelessness in managing work or performing a job effectively [27]. In this study, it was evaluated using 10 questions inquiring how frequently the medical students experienced various emotions related to burnout, with five possible responses: 1 (never), 2 (seldom), 3 (sometimes), 4 (often), and 5 (always) [27]. A confirmatory factor analysis was used to verify the items (*p* < .05) with an asymptotically distribution-free bootstrap model [21, 22] for construct validity testing and yielded moderate model fit. The Cronbach’s alpha was 0.854. One burnout score of 10 items was calculated by the mean for further analysis.

Compassion satisfaction refers to positive feelings about people’s ability to help and is characterised by a feeling of satisfaction with one’s job and from helping itself, feeling invigorated by the work that one likes to do, feeling that one can keep up with new technology and protocols, experiencing positive thoughts, feeling successful, being happy with the work one does and wanting to continue doing it, and believing that what one does makes a difference [27]. In this study, it was evaluated using 10 questions inquiring how frequently the medical students experienced various emotions related to compassion satisfaction, with five possible responses: 1 (never), 2 (seldom), 3 (sometimes), 4 (often), and 5 (always) [27]. A confirmatory factor analysis was used to verify the items (*p* < .05) with an asymptotically distribution-free bootstrap model [21, 22] for construct validity testing and yielded good model fit. The Cronbach’s alpha was 0.960. One compassion satisfaction score of 10 items was calculated by the mean for further analysis.

Detailed information is presented in Table 1.

#### Medical student demographics

The medical students’ sex and age were included as partially confounding factors for training stress [15] and resilience [26].

### Data collection

The medical students first completed a baseline survey that collected their demographic information and measured their resilience at the beginning of the clerkship. Subsequently, they regularly answered our surveys at each specialty rotation, providing their self-perceptions of training stress and professional quality of life. Because all of the medical students were free to decide whether to complete our surveys each time, each participant presented various responses during the study period. The medical students who submitted at least three responses to complete our individual rotating specialty surveys were included in this study. In total, 93 medical students with a total of 1073 responses were included.

### Statistical analysis

Descriptive analyses were performed to examine the medical students’ demographics, level of resilience, and perceived training stress and professional quality of life during their clerkships. It might be argued that the data in this study could be analysed using a two-level or multilevel analysis if variations were present in the medical students’ repeated responses regarding their burnout and compassion satisfaction at various specialty rotations with respect to their demographics and contexts. However, the intraclass correlation coefficients (ICCs) for the medical students’ burnout (ICC = 0.00259) and compassion satisfaction (ICC = 0.00356) at individual specialty rotations were < 0.05, the minimum cutoff point to perform multilevel analysis [28, 29]. Thus, multilevel effects were ignored in this dataset.

The 1073 responses to the individual rotating specialty surveys from the 93 medical students was set as the unit of analysis by which to examine the effects of perceived training stress as risk factors on professional quality of life, and the moderating effect of the medical students’ resilience was verified through hierarchical regressions [30, 31]. Two hierarchical regressions were performed for each of the dependent variables, namely burnout and compassion satisfaction. For each hierarchical regression, the medical students’ personal characteristics such as sex and age were entered into the model at the first step. The risk factors of the medical students’ perceived training stress, measured by psychological and physical demands (factor scores), were included at the second step. At the third step, the medical students’ resilience, recalculated by mean-centred for ensuring the normality of distribution, was entered as a protective factor to test its direct effect. Finally, at the fourth step, the interaction terms of the medical students’ resilience (mean-centred) and perceived psychological and physical demands in training (factor scores) were included. All analyses were performed using IBM SPSS (version 18.0).

## Results

Ninety-three medical students (43 men [46%] and 50 women [54%]; average age: 23 years) were included in our study. The medical students’ average resilience score measured at the beginning of their clerkships was 5.494 on average (*SD* = 0.735), with the items ‘I usually take things in stride’ and ‘I feel proud of having accomplished things in life’ having the lowest (mean = 4.000, SD = 1.460) and highest (mean = 6.151, SD = 0.955) scores, respectively. Detailed personal background information is listed in Table 1.

A review of the medical students’ 1073 responses to the individual specialty rotation surveys revealed that on average, the training stress perceived by the medical students was 2.696 (SD = 0.883) for psychological demands and 1.810 (SD = 0.819) for physical demands, whereas the average perceived professional quality of life was 2.346 (SD = 0.613) for burnout and 3.505 (SD = 0.815) for compassion satisfaction. Detailed descriptive analyses of the medical students’ perceived stress and professional quality of life on the basis of individual specialty rotations are presented in Table 2.

One hierarchical regression (unit of analysis = 1073 individual specialty rotation survey responses) was performed to test the buffering effect of the medical students’ resilience on the influence of stress on burnout during clinical training. The results revealed that the medical students’ perceived training stress, assessed in terms of psychological (β = 0.234, *p* < .001) and physical (β = 0.394, *p* < .001) demands, was related to increased burnout (Hypothesis 1, Step 2, Table 3) and that their resilience had a direct effect on reducing that burnout (β = − 0.350, *p* < .001) (Step 3, Table 3). Moreover, the results indicated that the medical students’ resilience buffered the negative effects of perceived training stress on their burnout in terms of physical (β = 0.104, *p* < .001) but not psychological (*p* > .05) demands (Hypothesis 2, Step 4, Table 3).

**Table 3:** Hypothesis testing for the buffer effect of the medical students’ resilience on their workplace stress and burnout during their clerkship (*n* = 1073)

Variables	Step 1	Step 2	Step 3	Step 4
Std ß				
Demographics				
Sex (default: male)	0.049	0.047	−0.018	- 0.025
Age	- 0.096**	−0.075**	−0.018	- 0.012
Workplace training stress				
Psychological demands (DM)	**–**	0.234***	0.204***	0.199***
Physical demands (DP)	**–**	0.394***	0.342***	0.351***
Resilience (RS)	**–**	**–**	−0.350***	- 0.361***
Buffering effect: interaction terms				
DM*RS	**–**	**–**	**–**	0.021
DP*RS	**–**	**–**	**–**	0.104***
R^2^	**0.012**	**0.222**	**0.332**	**0.344**

**p < 0.01; ***p < 0.001

Another hierarchical regression (unit of analysis = 1073 individual specialty rotation survey responses) was performed to test the buffering effect of the medical students’ resilience on the influence of stress on their compassion satisfaction during clinical training. The results revealed that the medical students’ perceived training stress was associated with a lower degree of compassion satisfaction in terms of physical (β = − 0.226, *p* < .001) and psychological (β = − 0.072, *p* < .05) demands (Hypothesis 1, Step 2, Table 4). In addition, the medical students’ resilience had a direct effect on increased compassion satisfaction (β = 0.384, *p* < .001; Step 3, Table 4) and could buffer the negative effects of perceived training stress on their compassion satisfaction with regard to physical (β = − 0.092, *p* < .01) but not psychological (*p* > .05) demands (Hypothesis 2, Step 4, Table 4).

**Table 4:** Hypothesis testing for the buffer effect of the medical students’ resilience on their workplace stress and compassion satisfaction during their clerkship (*n* = 1073)

Variables	Step 1	Step 2	Step 3	Step 4
Std ß				
Demographics				
Sex (default: male)	- 0.201***	- 0.199***	- 0.127***	- 0.119***
Age	0.023	0.010	- 0.053	- 0.055*
Workplace training stress				
Psychological demands (DM)	**–**	- 0.072*	- 0.039	- 0.040
Physical demands (DP)	**–**	- 0.226***	- 0.169***	- 0.178***
Resilience (RS)	**–**	**–**	0.384***	0.393***
Buffering effect: interaction terms				
DM*RS	**–**	**–**	**–**	0.020
DP*RS	**–**	**–**	**–**	- 0.092**
R^2^	**0.041**	**0.098**	**0.233**	**0.242**

*p < 0.05; **p < 0.01; ***p < 0.001

## Discussion

In this prospective study, 1073 questionnaire responses from 93 medical students undergoing their 1-year clerkship were analysed to determine the negative effects of perceived training stress on burnout and compassion satisfaction (Hypothesis 1). In addition, resilience was observed to moderate the physical demands that impacted the medical students’ professional quality of life, but not the psychological demands (Hypothesis 2).

Our study confirmed the effect of training stress on increased medical student burnout, as reported in other studies [7, 15, 32]. In particular, our study revealed that the medical students’ physical demands (β = 0.394, *p* < .001) had a larger effect on their burnout risk than did their psychological demands (β = 0.234, *p* < .001; Table 3). Physical demands were measured by four items, namely awkward arm position, awkward body position, rapid physical activity, and lifting a heavy load. Based on our findings regarding the challenging physical demands that medical students face, which were skills-oriented and had a substantial influence on the medical students’ risk of burnout during clerkship, we suggest that further efforts should be made to explicitly target the needs of medical students with clear objectives and learning activities tailored to them, as well as student and course evaluations on a cycle-by-cycle basis for continuous teaching improvement and support [33].

Psychological demands were measured by six items, namely being demanded to hard work, a fast-paced work environment, being busy at work, a lack of sleep, work fatigue, and intense work concentration. Studies have found that the main stresses for medical students during their clerkships are applying clinical knowledge, learning experientially, using clinical skills, adjusting to clinical settings, and understanding roles [32, 33], as well as trauma exposure [34]. A supportive learning environment, including social supports, can have both direct [7] and moderating effects on the negative influence of stress on medical students’ mental health [35]. Supervision refers to the monitoring, guidance, and feedback by physicians in the patient care setting and is performed to ensure individual, professional, and educational development [36]. We might argue that given the psychological demands on medical students, adequate supervision by physicians or experts in various specialties is crucial to students’ successful clinical learning with regards to thinking patterns, decision-making protocols, and activities involved in patient care. Supervision would help medical students learn efficiently and effectively and would alleviate the effects of the mental demands required in their workplace [37].

In this study, compassion satisfaction referred to how the medical students perceived their training as medical doctors and how satisfied they felt about this profession during their clerkships. Our study revealed that the physical (β = − 0.226, *p* < .001) and psychological (β = − 0.072, *p* < .05) demands perceived by the medical students were related to reduced compassion satisfaction. Studies have revealed that physical demands resulting in physical complaints are made by physicians of both surgical and nonsurgical specialties [38]. In addition, a population-based 7-year longitudinal follow-up study in Denmark revealed that high physical work demands had an effect on subsequent unemployment, sick leave, and permanent work disability [39]. We might argue that medical students, as novices in medical practice, experience greater physical demands resulting from their lack of efficiency or familiarity with the workload, leading to frustration in learning and reducing their compassion satisfaction. Skill-based clinical apprentice training, covering the cognitive phase through various pedagogical approaches (e.g. instructive training, case studies, video teaching, and clinical practice) [40], followed by the psychomotor phase whereby learners practice their skills [41, 42], should be emphasised. Moreover, medical students should be assisted in cultivating procedural and technical skills to overcome the physical demands of clinical training, including planning, procedural demonstrations, learner observation, feedback, self-assessment, practice, and approach modification [43].

In this study, the medical students’ baseline resilience was measured at the beginning of their clerkship. Our results revealed that resilience was related to reduced burnout and increased compassion satisfaction during clinical training (Step 3, Tables 3 and 4). Resilience has been proposed as a valuable construct underpinning positive coping strategies for learning and professional practice [44]. A conceptual model coined as ‘the coping reservoir’ was proposed to encompass the inputs gained by students practising medicine [13], including educational and environmental supports (e.g. psychosocial support, social and health activities, and mentorship) [13], as well as mindfulness-based stress-reducing interventions [32, 45]. All of these are key issues that should be further explored when studying the determinants of medical students’ resilience.

Here, our examination of the buffering role of resilience on medical students’ stress on their professional quality of life (Step 4, Tables 3 and 4) revealed that the buffering effects of resilience were observed for physical demands (one risk factor for the medical students’ professional quality of life), but not for psychological demands (another risk factor). Resilience is described as an adaptive capability that originates from the interaction between individuals and their immediate environments [46–48]. Notably, the physical demands measured in our study were more task-, technical-, or skill-related, and it might have been easier for the students to ‘bounce back’ from these demands in the short run (i.e. during their first year of clerkship), than it might be for them to adapt to the psychological demands in the long run. Future longitudinal tracing research should be conducted to explore this. Future studies could also focus on strategies based on the identified influencers to assist medical students in clerkships for psychological demands.

Regarding the determinants of the medical students’ burnout and compassion satisfaction results, we found that changes in R square (∆R^2^) values for burnout determinants from Steps 1 to 4 demonstrated that training demands are a risk factor (Steps 1 and 2, ∆R^2^ = 0.210) that explain a larger variance than does resilience as a protective factor (Steps 2 and 3, ∆R^2^ = 0.110) in workplace training during clerkship (Table 3). However, changes in ∆R^2^ values for compassion satisfaction determinants from Steps 1 to 4 showed that resilience is a protective factor that explains a larger variance (Steps 2 and 3, ∆R^2^ = 0.135) than does training stress as a risk factor (Steps 1 and 2, ∆R^2^ = 0.057) in workplace training during clerkship (Table 4). With our limited data, different mechanisms (predictive paths) leading to medical students’ professional quality of life such as burnout and compassion satisfaction would be expected for additional studies in the future.

Several limitations of this study must be mentioned. First, only one cohort of medical students from one medical school was examined. Second, the stress experienced by the medical students in clinical training was measured according to workplace training demands. However, the sources of stress may vary and include problems such as poor team dynamics, witnessing a patient’s suffering or death, personal mistreatment, or poor role modelling by physicians [14, 34]. Third, the resilience level of medical students was measured at the beginning of their clerkships, whereby it was implicitly assumed that resilience would remain stable. However, resilience is variable and achievable [49] and serves as a criterion for self-adaptability [50]. Future studies could thus explore the dynamics of resilience levels in medical students. Supportive learning environments may have positive effects on the development of resilience among medical students in clinical training [51]. Moreover, various cultural norms could be an issue when identifying factors related to medical students’ resilience [52, 53].

## Conclusions

With a prospective cohort design, this study analysed 1073 questionnaire responses of 93 medical students during their 1-year clerkship. Our study verified the negative effects of the students’ perceived training stress on burnout and compassion satisfaction. While resilience was the moderate the effects of physical demands (one risk factor for the medical students’ professional quality of life), it did not moderate the effects of psychological demands (another risk factor). Moreover, different mechanisms (predictive paths) leading to medical students’ professional quality of life such as burnout and compassion satisfaction warrant additional studies.

## Data Availability

The datasets used for the current study are available from the corresponding author on reasonable request.

## Abbreviations

AGFI: Adjust Goodness of Fit Index
CFI: Comparitive Fit Index
CR: Critical ratio
GFI: Goodness of Fit Index
ICC: Intraclass Correlation Coefficient
IFI: Incremental Fit Index
ProQOL: Professional Quality of Life Scale
RMSEA: Root Mean Square Error of Approximation
SD: Standard Deviation
TLI: Tucker-Lewis Index
